# Integrated Transcriptomic Analysis of Liver and Muscle Tissues Reveals Candidate Genes and Pathways Regulating Intramuscular Fat Deposition in Beef Cattle

**DOI:** 10.3390/ani15091306

**Published:** 2025-04-30

**Authors:** Siwei Wang, Tingting Liu, Peng Peng, Yurong Fu, Shaoqing Shi, Shuang Liang, Xi Chen, Kun Wang, Rongyan Zhou

**Affiliations:** 1 College of Animal Science and Technology, Hebei Agricultural University, Baoding 071000, China; wangsiwei0909@126.com; 2 Key Laboratory of Crop Cultivation Physiology and Green Production of Hebei Province, Institute of Cereal and Oil Crops, Hebei Academy of Agriculture and Forestry Sciences, Shijiazhuang 050035, China; 15930885886@163.com (T.L.); pengp1994@163.com (P.P.); fuyurong2023@163.com (Y.F.); shisq1991@163.com (S.S.); wyoubbjmeefn@gmail.com (S.L.); chenx202012@163.com (X.C.)

**Keywords:** intramuscular fat, beef cattle, transcriptomic, *longissimus dorsi* muscle, liver

## Abstract

Intramuscular fat (IMF) content in beef cattle is a critical determinant of beef meat quality, as it positively influences juiciness, tenderness, and palatability. In China, the crossbreeding of Wagyu and Angus is a prevalent method for achieving a better marbling level. However, the molecular mechanisms governing IMF regulation in these crossbreeds remain poorly understood. To elucidate the mechanism of IMF deposition in these crossbred cattle, we conducted a comparative transcriptomic analysis of *longissimus dorsi* muscles and livers from cattle with divergent IMF content. RNA-seq revealed 940 and 429 differentially expressed genes (DEGs) in the liver and muscle, respectively, with 60 genes co-differentially expressed (co-DEGs) in both tissues. Functional enrichment highlighted lipid metabolism pathways including fatty acid β-oxidation, PPAR signaling, and glycerolipid metabolism. A total of eleven genes including *ACAA2*, *ACADL*, *ACOX2*, *CPT1B*, *CPT2*, *LPL*, *SLC27A1*, *ACAT1*, *GK*, *ACOX3*, and *ACSM5*, were screened as key candidate genes for IMF deposition. A “liver–muscle” regulatory network of IMF deposition was built to illustrate the tissues’ interaction. The reliability of the transcriptomic data was verified by quantitative reverse real-time PCR (qRT-PCR). Our findings provide novel molecular markers for increasing the IMF content and accelerating the genetic improvement of beef quality traits in crossbred cattle.

## Simple Summary

Improving beef marbling is critical for meeting market demands. Still, it remains difficult due to the complicated biological processes of intramuscular fat deposition. While crossbreeding Wagyu and Angus cattle becomes popular in China to produce premium marbled beef, the genetic mechanism behind this process remains unclear. To elucidate the complex mechanisms of intramuscular fat deposition, we performed transcriptomic analyses of liver and muscle tissues from Wagyu–Angus crossbred cattle with differing intramuscular fat levels. Analysis revealed over 1300 differentially expressed genes, with sixty genes exhibiting differential expression in both tissues. Further analysis identified eleven key candidate genes as potential regulators, and the PPAR signaling pathway and fatty acid β-oxidation pathway play important roles in this process. Finally, we constructed a “liver–muscle” regulatory network of IMF deposition to illustrate the tissues’ interaction. Our findings provide valuable genetic markers for selectively breeding cattle with higher IMF content, improving beef quality, and increasing farmers’ financial advantages.

## 1. Introduction

The rapid economic growth and urbanization in China has elevated consumer demand for premium beef characterized by superior sensory attributes (e.g., tenderness, juiciness) and nutritional value [1]. Therefore, breeding efforts for beef cattle now prioritize improving the quality of the meat, particularly with respect to intramuscular fat (IMF) content. IMF—which consists of lipid droplets within muscle cells and interspersed adipose tissue among muscle fibers [2]—deposition occurs predominantly during the late fattening phase, making it one of the most metabolically challenging and economically costly traits to optimize [3]. However, the IMF adipocytes, possessing smaller size and higher unsaturated fatty acids content compared to other adipose tissues, play a pivotal role in enhancing the meat’s flavor, juiciness, and tenderness [4,5,6], thereby amplifying the market value of high-marbled beef. Consequently, deciphering the molecular regulation of IMF deposition has become a central focus for improving both meat quality and production efficiency.

The synthesis of IMF is regulated by various factors, including genetics, breed, management, and nutrition [7], with the breed being the most critical. Among various breeds, Wagyu cattle have the highest IMF content, reaching 36.5% in the *longissimus dorsi* (LD) muscle, followed by Korean cattle at 13.7%, and Angus at 9.3% [7]. The deposition of intramuscular fat is a complex process that involves a highly coordinated program of gene expression. Numerous molecules have been identified as playing key roles in regulating this formation process, including candidate genes represented by *FABPs* [8,9], *AdipoQ* [10,11], *MC4R* [12], *LPL* [13], *SCD* [14], and *FASN* [15]. Additionally, this process is significantly influenced by transcription factors that regulate adipocyte differentiation, including the PPAR family [16], C/EBP family [17], RAR family [18], and SREBP family [14,19], as well as non-coding RNAs (ncRNAs) that participate in regulation [20,21,22,23]. Furthermore, the application of high-throughput transcriptome sequencing has significantly advanced the understanding of the regulatory mechanisms affecting IMF in cattle. Yu et al. conducted RNA sequencing on Qinchuan, Nanyang, and Japanese black cattle, uncovering that *ITGB1* serves as the essential gene linked to IMF content [24]. Meanwhile, Wang et al. employed whole transcriptome sequencing examining seven different tissues from Jiaxian red cattle to elucidate the complexities of fat deposition processes [25]. Experiences in production indicate that cattle of the same breed when raised in identical environments and fed the same diet, can exhibit markedly different levels of IMF. This variation suggests that the genetic potential of each individual can have a substantial impact on the deposition of IMF [26]. However, most research has focused on inter-breed differences, leaving the genetic mechanisms underlying IMF deposition within specific breeds unclear, and the effective molecular markers for improving IMF content in cattle remain to be fully explored and require further investigation.

In China, due to restrictions on the import of Wagyu cattle, crossbreeding Australian Wagyu with Angus cattle has become the most common way to produce high-quality meat. However, research on the IMF regulation in the crossbred remains limited. Furthermore, being the primary metabolic organ, the liver is critical to lipid metabolism and is actively involved in fatty acid synthesis, degradation, and transport, as well as lipoprotein assembly and secretion [27,28]. Studying the liver alongside muscle could help clarify the mechanism of IMF deposition. Therefore, we employed RNA-Seq technology to conduct comparative transcriptomics analysis on the LD muscle and liver tissues of Wagyu and Angus crossbred individuals with significantly different IMF content. Moreover, we also studied the cellular morphology of myocytes and adipocytes with different IMF content. Our research aims to pinpoint genes that are differentially expressed (DEGs) and the pathways linked to the regulation of IMF, which could provide valuable molecular markers for increasing IMF content in beef and accelerating genetic improvement of beef quality.

## 2. Materials and Methods

### 2.1. Animals and Sample Collection

Wagyu × Angus crossbred F1 bulls (n = 26) were maintained under uniform housing and dietary conditions and slaughtered at 28 months of age with an average carcass weight of 324.0 ± 17.66 kg. Animals were fed a Total Mixed Ration (TMR) formulated to meet NRC (2016) [29] requirements for finishing beef cattle. The diet contained 55% corn, 15% alfalfa hay, 12% soybean meal, 4% fat powder (dry matter basis) and so on, providing 14.0% crude protein and 12.6 MJ/kg metabolizable energy. Full dietary composition and nutritional profiles are detailed in Supplementary Table S11. The procedures for slaughtering and sampling followed the established guidelines for the care and use of experimental animals. The experiments were conducted following the Regulations governing the Administration of Affairs Related to Experimental Animals and received approval from the Animal Research Committee at Hebei Agricultural University (approval number: 2024170; date of approval: 13 December 2024).

After slaughter, liver and LD muscle samples at the 12–13th rib were collected, trimmed to remove external fat and connective tissue, and portioned for IMF quantification, hematoxylin-eosin (HE) staining, and RNA extraction. The samples designated for IMF measurement were stored in frozen CO_2_ and then at −20 °C until use; the samples meant for HE staining were kept in test tubes with Tissue Fixation Solution (Servicebio, Wuhan, China), while the samples for RNA extraction were rapidly frozen in liquid nitrogen and maintained at −80 °C until required.

### 2.2. IMF Measurements

The content of IMF in LD muscle samples was measured using the Soxhlet extraction technique. First, meat tissues were pulverized using a meat grinder, then about 3 g of meat was weighed in each bottle and hydrolyzed using hydrochloric acid (100 mL, 2 mol/L) in a water bath maintained at 80 °C for 1 h, followed by drying at 105 °C for 1 h. An extraction solution of petroleum ether, boiling between 30 and 60 °C, was employed, with the samples extracted at 60 °C for 5 h. After extraction, the samples were heated and dried for 2 h. Subsequently, the samples were cooled to room temperature to measure the IMF content. The IMF content (%) was calculated using the following formula:

$$x=\frac{m1-m0}{m2}\times 100\%$$

m1 represents the weight of the receiving bottle with the extracted IMF (g), m0 signifies the receiving bottle’s net weight (g), and m2 indicates the weight of the tested sample.

### 2.3. HE Staining

Three LD muscle samples exhibiting high and low intramuscular fat content were selected. Following the procedure described by Matsunari et al. [30], tissue samples were fixed in Tissue Fixation Solution for 24 h to ensure consistent preservation across all samples. Paraffin slices (7 μm) were then produced and stained with HE (hematoxylin: 5 min; eosin: 4 min). Slides were scanned using an Aperio AT2 digital scanner (Leica Microsystems, Shanghai, China), and images were analyzed using Aperio ImageScope x64 software (version 12.4.6, Aperio Technologies, Vista, CA, USA). Measurements of diameter (longest axis for adipocytes; shortest axis for myocytes), perimeter (cellular boundary length), and area (two-dimensional projection) were obtained for at least 200 myocytes and adipocytes per section, from at least three sections per sample. Only round, intact cells were selected to minimize measurement error.

### 2.4. RNA Extraction, RNA-Seq Library Construction, and Sequencing

Based on the distribution of IMF values, samples were classified into high-IMF (n = 4, IMF content ≥ 25%) and low-IMF groups (n = 4, IMF content ≤ 15%). The threshold values for high and low IMF were determined based on the upper and lower quartiles of the IMF distribution, respectively. Samples were designated as high liver (HL), high muscle (HM), low liver (LL), and low muscle (LM) groups. Total RNA was extracted using Trizol^®^ reagent (Invitrogen, Waltham, MA, USA), and its integrity was assessed by agarose gel electrophoresis and NanoDrop spectrophotometry (RIN ≥ 7.0). A total of 3 μg of total RNA was used to purify mRNA using poly-T magnetic beads. DNA libraries were then prepared through fragmentation, cDNA synthesis, end-repair, A-tailing, and adapter ligation using Illumina PE adapters (Illumina, San Diego, CA, USA). Libraries were size-selected (400–500 bp) with AMPure XP beads (Beckman Coulter, Brea, CA, USA), amplified by 15 cycles of PCR using Illumina PCR Primer Cocktail (Illumina, San Diego, CA, USA), purified again with AMPure XP beads, and quantified using an Agilent Bioanalyzer 2100 system (Agilent Technologies, Santa Clara, CA, USA). The final libraries were sequenced at 150 bp bipartite on the NovaSeq 6000 platform (Illumina, San Diego, CA, USA) (Shanghai Parsonage Biotechnology Co., Ltd., Shanghai, China). The raw sequencing data have been submitted to the NCBI database (accession number: PRJNA1225315).

### 2.5. Transcriptome Data Processing and Differential Gene Expression Analysis

Raw sequencing reads (fastq format) were processed to obtain clean reads using fastp (v0.22.0). Clean reads were then mapped to the ARS-UCD2.0 bovine reference genome (https://www.ncbi.nlm.nih.gov/datasets/genome/GCF_002263795.3/ (accessed on 9 September, 2024)) using HISAT2 (v2.1.0). Read counts per gene were quantified using HTSeq (v0.9.1) and normalized to FPKM (Fragments Per Kilobase Million). Differentially expressed genes (DEGs) were identified using DESeq2 (v1.38.3) with thresholds of |log2FoldChange| > 1 and *p*-value < 0.05. ComplexHeatmap (v2.16.0) was used for bi-directional hierarchical clustering of samples based on gene expression, using Euclidean distance and complete linkage.

### 2.6. Functional Enrichment and Network Analysis

Gene Ontology (GO) enrichment analysis was performed using ClusterProfiler (v4.6.0) to identify significantly enriched GO terms (*p* < 0.05), providing insights into the biological functions of DEGs. Kyoto Encyclopedia of Genes and Genomes (KEGG) pathway enrichment analysis was also conducted using ClusterProfiler (v4.6.0), focusing on pathways with *p* < 0.05. Protein–protein interaction (PPI) network analysis was performed using the STRING database (accessed on 21 January 2024) to reveal relationships between target genes. The resulting PPI network was visualized using Cytoscape (version 3.8.0), and plots were generated using the CNSknowall platform (https://cnsknowall.com (accessed on 13 November 2024)).

### 2.7. qRT-PCR Validation of RNA-Seq Results

To validate the RNA-Seq results, we performed qRT-PCR on 14 selected genes. Total RNA was reverse transcribed using the PrimeScript™ FAST RT reagent Kit. Gene expression (*ACSL4*, *IRF7*, *HRG*, *PLTP*, *CD74*, *HK2*, *GPAM*, *ACOX3*, *LIPE*, *LPL*, *CPT1B*, *EBPL*, *RET*, and *PARP14*) was quantified using a CFX Opus 384 Real-Time PCR System with *ACTB* as a reference gene. Primers were designed using Primer-BLAST (Supplementary Table S1, https://www.ncbi.nlm.nih.gov/tools/primer-blast/, accessed on 22 January 2024). qRT-PCR was performed with a 10 μL reaction volume with 5 μL TB Green^®^ Premix Ex Taq™ II FAST qPCR (Takara, Shiga, Japan), 0.2 μL of 10 μM primers each, 3 μL of cDNA dilution 1:5, and 1.6 μL DNase-RNase Free H_2_O, and thermal cycling conditions included 40 cycles of 95 °C for 5 s and 60 °C for 30 s. Primer efficiency ranged from 1.87 to 2.00 (R^2^ ≈ 0.99). Gene expression was calculated using the 2^−∆∆Ct^ method.

### 2.8. Statistical Analysis

Statistical analyses were performed using SPSS (version 22.0; IBM Corp.) and GraphPad Prism (version 10.0; GraphPad Software). Data were tested for normality (Shapiro–Wilk) and homogeneity of variances (Levene’s test). Group comparisons were conducted using unpaired *t*-tests (*p* < 0.05). Pearson correlation coefficients (*r*) were calculated to evaluate associations between gene expression and IMF content, with *p*-values adjusted for multiple comparisons (Benjamini–Hochberg FDR). A priori power analysis (G*Power, version 3.1.4) confirmed adequate sample size (α = 0.05, power = 0.8, Cohen’s d = 1.2). Data are presented as mean ± SD.

## 3. Results

### 3.1. Differences in Meat Quality of Cattle with Different IMF Contents

Four samples with a high level of IMF and four with a low level were selected from the collected samples, ensuring similar carcass weights (*p* > 0.05). These were grouped as high and low IMF groups and analyzed for slaughtering performance and meat quality traits (Table 1). The high IMF group exhibited significantly higher IMF content (29.02 ± 6.28% vs. 11.70 ± 1.10%, *p* = 0.01) and marbling score (*p* < 0.01) than the low IMF group. Differences in fat and meat color were not statistically significant (*p* > 0.05).

### 3.2. Morphological Differences Among LD Muscle with Different Levels of IMF Content

**Figure 1:**
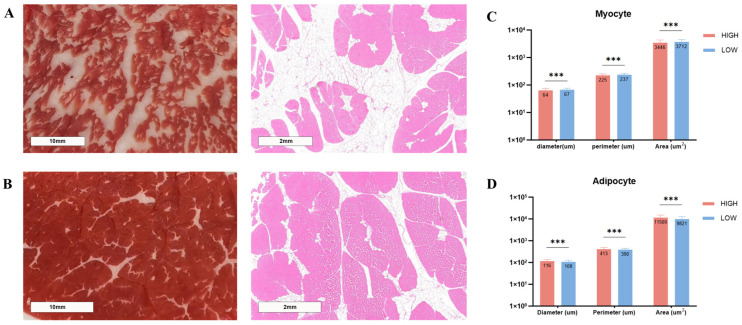
Histological analysis of *longissimus dorsi* (LD) muscle in (**A**) high and (**B**) low intramuscular fat (IMF) groups. Left panels: photographs of LD muscle samples; right panels: HE-stained sections of LD muscle with scale bars indicating 2 mm. Morphometric measurements of (**C**) myocytes and (**D**) adipocytes. The asterisks indicating significance level: *** *p* < 0.001.

HE staining was applied to LD muscles between HM and LM groups and revealed a marked difference in fat distribution (Figure 1). The high IMF group (Figure 1A) showed substantially greater intramuscular adipocyte density than the low IMF group (Figure 1B), confirming the previously determined IMF content. According to morphometric analysis, myocytes in the HM group had significantly reduced cell diameter, perimeter, and area (*p* < 0.01) (Figure 1C), while adipocytes were significantly larger (*p* < 0.01) than those in the LM group (Figure 1D).

### 3.3. Summary of RNA-Seq Results and Identification of DEGs

After processing 16 samples for transcriptome sequencing, 115.17 Gb of clean data were produced. The RNA-Seq data are summarized in Supplementary Table S2. At least 6.32 Gb of clean data were generated for each sample, and the quality score of Q30 was achieved from 92.86% to 96.44%. Clean reads from each sample were mapped to the reference genome ARS-UCD2.0. The mapping ratio of each sample against the reference genome ranged from 91.49% to 98.44%.

**Figure 2:**
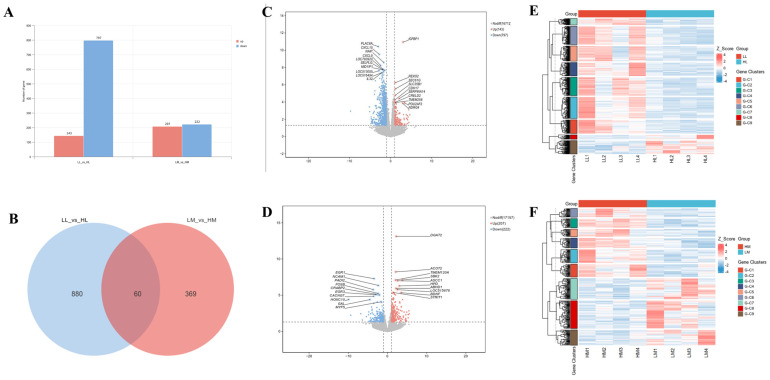
Differential gene expression (DEG) and clustering analysis in liver and muscle with varying intramuscular fat (IMF) content. (**A**) Number of DEGs; (**B**) Venn diagram of shared DEGs; Volcano plots in (**C**) liver (LL vs. HL) and (**D**) muscle (LM vs. HM); Hierarchical clustering of gene expression in (**E**) liver and (**F**) muscle.

DEG analysis (*p* < 0.05, |log2FC| ≥ 1) identified 940 DEGs in the liver (LL vs. HL) and 429 DEGs in the muscle (LM vs. HM) (Figure 2A, Supplementary Tables S3 and S4), with 60 genes co-differentially expressed (co-DEGs) in both tissues (Figure 2B, Supplementary Table S5). Specifically, HL showed 143 upregulated and 797 downregulated genes compared to LL, while HM showed 207 upregulated and 222 downregulated genes compared to LM. Notable DEGs included *IGFBP1*, *REXO2* (upregulated), *PLAC8A*, *CXCL10*, *NAIP* (downregulated) in liver (Figure 2C), and *DGAT2*, *ACOT2*, *ABHD1* (upregulated), *EGR1*, *NCAM1*, *MYF5* (downregulated) in muscle (Figure 2D). The z-score-transformed gene expression data were also subjected to unsupervised hierarchical clustering using Euclidean distance with complete linkage. K-means partitioning (k = 9) successfully clustered liver and muscle samples into distinct groups based on IMF contents, supporting the existence of tissue-specific transcriptional programs regulating IMF deposition (Figure 2E,F).

### 3.4. Functional Enrichment Analysis of DEGs in Liver and Muscle Reveals Tissue-Specific Biological Processes Associated with Intramuscular Fat

#### 3.4.1. GO Term Enrichment in Liver and Muscle DEGs Related to Intramuscular Fat

**Figure 3:**
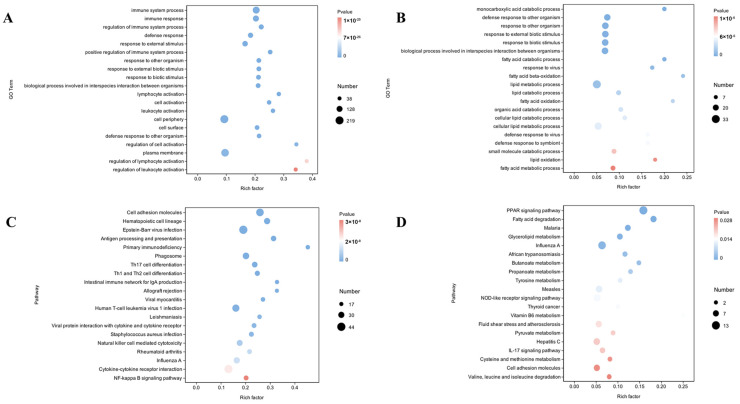
Functional annotation analysis of DEGs among different groups: (**A**) top 20 Gene Ontology (GO) pathways in the liver; (**B**) top 20 GO pathways in muscle; (**C**) top 20 Kyoto Encyclopedia of Genes and Genomes (KEGG) pathways in the liver; (**D**) top 20 KEGG pathways in muscle.

To better understand the biological mechanisms between different IMF content, GO enrichment analyses of the DEGs in the liver and muscle, which included biological process (BP), cellular component (CC), and molecular function (MF) enrichment were conducted. A total of 928 GO terms (*p* < 0.05) were considerably enriched in the liver’s DEGs, with 667 fitting into the BP category, 56 into the CC category, and 205 into the MF category. These terms were primarily enriched in the immune system process, immune response, and regulation of the immune system process, etc. (Figure 3A, Supplementary Table S6). At the same time, DEGs in the muscle were significantly enriched in 546 GO terms (*p* < 0.05), including 388 in the BP category, 32 in the CC category, and 126 in the MF category. These GO terms were primarily related to the monocarboxylic acid catabolic process, fatty acid catabolic process, fatty acid beta-oxidation, and lipid metabolic process (Figure 3B, Supplementary Table S7).

#### 3.4.2. KEGG Pathway Enrichment in Liver and Muscle DEGs Related to Intramuscular Fat

A KEGG enrichment analysis was performed to investigate the biological roles of the DEGs. There was a substantial (*p* < 0.05) enrichment of 65 KEGG pathways in the liver and 27 in the muscle. In the liver, the DEGs were significantly enriched in cell adhesion molecules, hematopoietic cell lineage, phagosome, etc. (Figure 3C, Supplementary Table S8). In addition, there was a notable enrichment of DEGs in muscle in functions involving the PPAR signaling pathway, fatty acid degradation, and glycerolipid metabolism (Figure 3D, Supplementary Table S9).

### 3.5. Candidate Genes in Liver-Muscle Interaction for IMF Deposition

**Figure 4:**
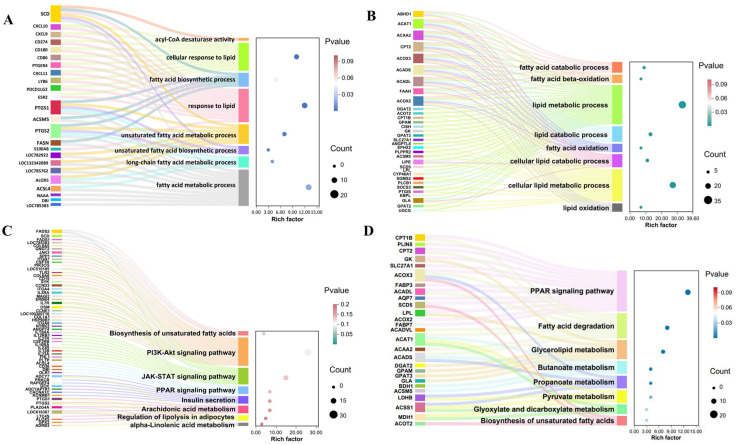
Lipid metabolism pathways in liver and muscle tissue: (**A**) GO terms in liver tissue; (**B**) GO terms in muscle tissue; (**C**) KEGG pathways in liver tissue; (**D**) KEGG pathways in muscle tissue.

**Figure 5:**
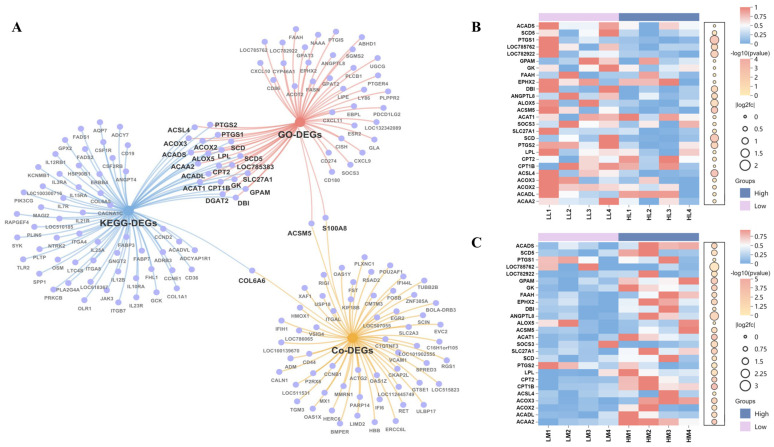
Overlapping genes and expression profiles: (**A**) Venn diagram of overlapping DEGs, GO−DEGs, and KEGG−DEGs; (**B**) heatmap of overlapping gene expression in liver tissue; (**C**) heatmap of overlapping gene expression in muscle tissue.

**Figure 6:**
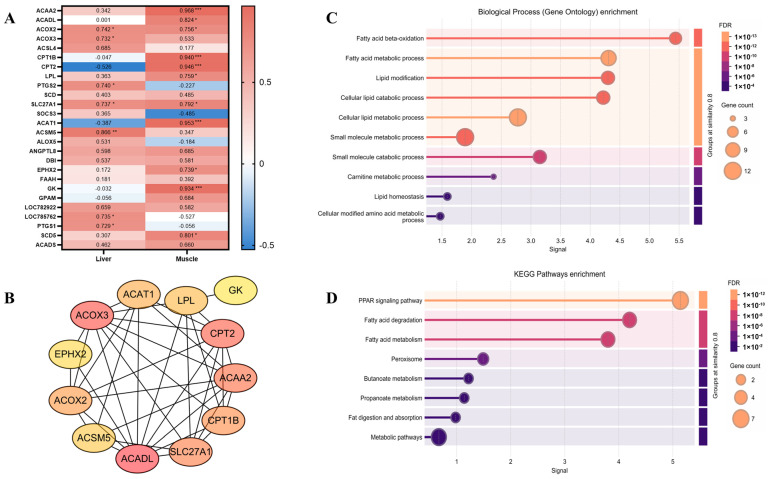
Identification of hub genes regulating IMF deposition. (**A**) Correlation analysis of gene expression and IMF content. Values represent Pearson’s correlation coefficients (*r*), with asterisks indicating significance levels: * *p* < 0.05, ** *p* < 0.01, *** *p* < 0.001. (**B**) Protein–protein interaction (PPI) network and ranking of hub genes. Node color intensity indicates ranking by the Maximal Clique Centrality (MCC) algorithm (darker color = higher rank). (**C**) GO enrichment analysis of biological processes for genes in cluster 1. (**D**) KEGG pathway enrichment analysis for genes in cluster 1.

To identify candidate genes involved in IMF deposition, eight pathways directly related to lipid metabolism were selected from GO and KEGG pathway analyses of liver and muscle tissues (Figure 4). A total of 57 genes were identified from GO pathways and 78 from KEGG pathways. These genes were enriched in pathways such as PPAR signaling, fatty acid degradation, unsaturated fatty acid biosynthesis, response to lipids, and lipid metabolic processes. A total of 26 genes were identified as overlapping between co-DEGs, GO-DEGs, and KEGG-DEGs (Figure 5A). The expression levels of these overlapping genes in each individual are summarized in Figure 5B,C. Subsequently, correlation analysis was performed between these genes and IMF content, and 16 genes with significant correlations: 11 in the muscle (*ACAA2*, *ACADL*, *ACOX2*, *CPT1B*, *CPT2*, *LPL*, *SLC27A1*, *ACAT1*, *EPHX2*, *GK*, *SCD5*), 7 in the liver (*ACOX2*, *ACOX3*, *PTGS2*, *SLC27A1*, *ACSM5*, *LOC785762*, *PTGS1*), and 2 (*ACOX2*, *SLC27A1*) in both tissues were identified (Figure 6A).

**Figure 7:**
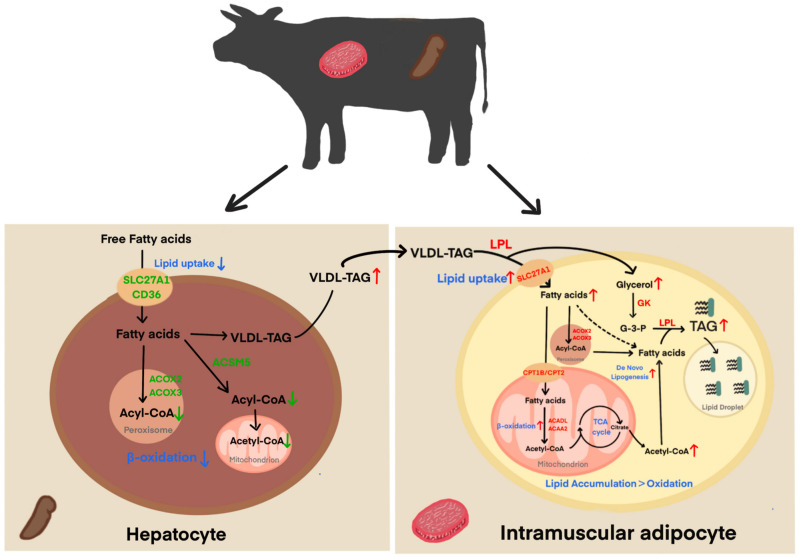
Mechanism regulating intramuscular fat deposition in the liver and LD muscle. Red nodes: upregulation; green nodes: downregulation. Abbreviations: *SLC27A1*: solute carrier family 27 member 1; *CD36*: cluster of differentiation 36; *ACSM5*: acyl−CoA synthetase medium chain family member 5; *ACOX2/3*: acyl−CoA oxidase 2/3; *LPL*: lipoprotein lipase; *CPT1B/2*: carnitine palmitoyltransferase 1B/2; *ACADL*: long−chain acyl−CoA dehydrogenase; *ACAA2*: acetyl−CoA acyltransferase 2; TCA cycle: tricarboxylic acid cycle; *VLDL*−*TAG*: very low−density lipoprotein−associated triacylglycerol; G−3−P: glycerol−3−phosphate.

To investigate functional relationships, 14 interacting genes were identified by PPI network analysis utilizing STRING. K-means clustering of these genes revealed two distinct clusters: cluster 1 (12 genes: *ACAA2*, *ACADL*, *ACOX2*, *CPT1B*, *CPT2*, *LPL*, *SLC27A1*, *ACAT1*, *EPHX2*, *GK*, *ACOX3*, *ACSM5*) and cluster 2 (2 genes: *PTGS1*, *PTGS2*). To prioritize potential regulators of IMF deposition, the 12 genes within cluster 1 were ranked using Cytoscape’s cytoHubba plugin and the Maximal Clique Centrality (MCC) algorithm (Figure 6B). Based on this ranking and their involvement in key metabolic pathways, a final set of 11 genes, including *ACAA2*, *ACADL*, *ACOX2*, *CPT1B*, *CPT2*, *LPL*, *SLC27A1, ACAT1*, *GK*, *ACOX3*, and *ACSM5*, were selected as key candidate genes for IMF deposition. Subsequent functional enrichment analysis indicated that these candidate genes were primarily involved in fatty acid β-oxidation and PPAR signaling pathways (Figure 6C,D). Then, A “liver-muscle” regulatory network of IMF deposition was constructed based on their expression levels in two tissues and function (Figure 7).

### 3.6. RNA-Seq Data Validation by qRT-PCR

**Figure 8:**
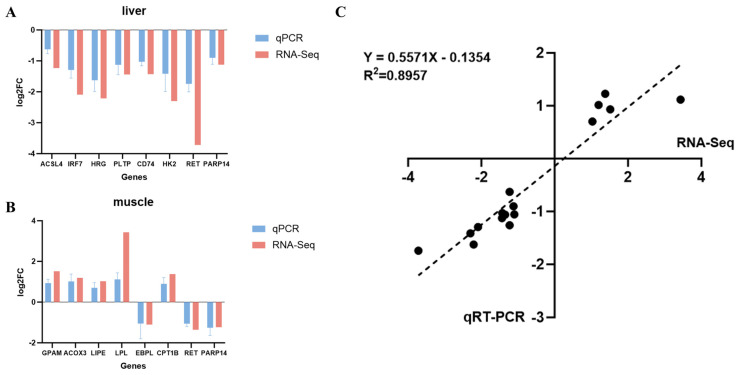
qRT−PCR validation of RNA−Seq data: (**A**) liver gene expression; (**B**) muscle gene expression; (**C**) Pearson correlation between qRT−PCR and RNA-Seq expression, each point represents a gene.

qRT-PCR was performed on six randomly selected DEGs from the liver, six from the muscle, and two co-DEGs to validate the RNA-Seq data. The expression patterns observed by RNA-Seq were consistent with qRT-PCR results across all genes, confirming the reliability of our methods (Figure 8). Specifically, in high vs. low IMF groups, the liver showed down-regulation of *ACSL4*, *IRF7*, *HRG*, *PLTP*, *CD74*, and *HK2*, while the muscle showed up-regulation of *GPAM*, *ACOX3*, *LIPE*, *LPL*, and *CPT1B*, and down-regulation of *EBPL*. *RET* and *PARP14* were downregulated in both the liver and the muscle. Pearson correlation analysis validated the consistency of qRT-PCR and RNA-Seq results (R^2^ = 0.8957).

## 4. Discussion

Numerous transcriptomic studies have investigated IMF deposition in cattle, but most have focused on identifying differentially expressed genes between breeds with differing IMF capacities [24,31,32]. In contrast, our study investigates intra-population variation in IMF within a commercially relevant crossbred population under controlled conditions. This approach overcomes the limitations of inter-breed comparisons, which often confound breed-specific genetic differences with the mechanisms of IMF deposition. By integrating comparative transcriptomics of LD muscle and liver tissues with analyses of cellular morphology, we provide a more nuanced understanding of the regulatory mechanisms driving differential IMF accumulation within this important crossbred population.

The integration of phenotypic and transcriptomic data provides a multi-level explanation for the divergent IMF deposition between groups. First, histological analysis revealed that high-IMF cattle exhibited significantly smaller myocytes (diameter: 32.5 ± 2.1 μm vs. 45.8 ± 3.4 μm, *p* < 0.01) and larger adipocytes (area: 1250 ± 150 μm^2^ vs. 780 ± 90 μm^2^, *p* < 0.01) compared to the low-IMF group (Figure 1C,D), similar results have been reported in pigs [33]. This morphological shift is consistent with the downregulation of myogenic regulators such as MYF5 (Figure 2D) and the upregulation of lipid storage genes such as LPL and CPT1B (Figure 6A), suggesting a potential transcriptional trade-off between myogenesis and adipogenesis. Second, the transcriptomic activation of PPAR signaling and fatty acid β-oxidation pathways (Figure 3D) supports enhanced lipid turnover in muscle, which directly correlates with the elevated IMF content (Table 1). Notably, the absence of adipocyte differentiation pathway enrichment (e.g., PPARγ, C/EBPα) in our data (Supplementary Table S7) indicates that the larger adipocyte size in high-IMF cattle is driven by lipid accumulation during fattening (consistent with [34]), rather than hyperplasia, which aligns with previous findings that adipocyte number is primarily determined during early development [35]. Finally, the coordinated downregulation of hepatic fatty acid oxidation genes such as *SLC27A1* facilitate the redirection of lipid substrates to the muscle, further amplifying intramuscular fat deposition. These findings collectively demonstrate that IMF variation in Wagyu–Angus crossbreeds is mechanistically linked to metabolic reprogramming favoring lipid storage over muscle hypertrophy.

Transcriptomic analysis reveals the PPAR signaling pathway and fatty acid β-oxidation pathway as key regulators of IMF deposition. The involvement of the PPAR signaling pathway in lipid metabolism has been established in various cattle species, such as Simmental and Pingliang Red cattle [36], Nanyang cattle, and Japanese black cattle [24], as well as in pigs [37] and chicken [38]. Lim et al. found differential expression of ten PPAR signaling pathway genes between high- and low-marbled Hanwoo cattle, with fatty acid oxidation identified as the primary downstream pathway [39], which was consistent with our research. We identified thirteen differentially expressed genes in the PPAR signaling pathway between HM and LM groups, while seven genes in the liver, including seven identified as key genes involved in IMF deposition, and most of them are also playing important roles in lipid metabolism, adipocyte differentiation, and gluconeogenesis processes. Among them, *SLC27A1*, which is also identified as *FATP1*, is notable for its critical role in fatty acid transport and metabolism. *SLC27A1* is an important membrane protein that promotes the absorption of long-chain fatty acids, which are essential for oleic acid synthesis in beef. It was reported that overexpression of *SLC27A1* in bovine preadipocytes promotes adipocyte differentiation, whereas interference with *SLC27A1* expression inhibits the process [40]. In addition, association studies have identified two SNPs in *SLC27A1* significantly impacting meat quality traits in Qinchuan cattle [41], highlighting its association with fat deposition. Our study provides further validation of the role of these genes and pathways.

The fatty acid β-oxidation pathway, which involves the breakdown of fatty acids for energy, plays a crucial role in regulating the balance between fat deposition and excretion, thereby influencing IMF content. Yang et al. found that DE circRNAs (DECs) and DE lncRNAs (DECs) were enriched for the GO pathway by whole transcriptome sequencing analysis of Qinchuan cattle intramuscular adipocytes at different differentiation stages, and both were significantly enriched in fatty acid β-oxidation pathway [42]. In our study, seven key genes were identified in this pathway, including *ACOX2*, *ACOX3*, *ACADL*, *CPT1B*, *CPT2*, *ACAT1*, and *ACAA2*. *ACOX2* and *ACOX3* are peroxisomal enzymes involved in the first step of the β-oxidation pathway with different substrate preferences, they catalyze the initial desaturation of acyl-CoAs, producing enoyl-CoAs [43]. It has been reported that *ACOX2* may be associated with the meat quality of yak [44], while *ACOX3* has been screened to be related to IMF in Qinchuan cattle [45] and chicken [46]. CPT1B and CPT2 facilitate fatty acyl-CoA conversion to acyl-carnitines, enabling fatty acid transport across the inner mitochondrial membrane and into peroxisomes for their subsequent oxidation [47]. Transcriptome analysis has revealed that *CPT1B* might significantly affect IMF deposition in Laiwu pigs, and *CPT2* might be involved in IMF regulation in Ningxiang pigs [48] and chicken [46]. *ACADL*, which encodes the mitochondrial enzyme LCAD that initiates long-chain fatty acyl-CoA oxidation [49], promotes goat intramuscular adipocyte differentiation through TNF signaling pathway activation [50]. *ACAT1*, encoding the mitochondrial enzyme that catalyzes the final step of long-chain fatty acid β-oxidation [51], has been identified as a candidate gene for the ribeye area trait in Nelore cattle via WGCNA [52]. ACAA2, a key enzyme responsible for the thiolytic cleavage of 3-oxoacyl-CoAs into acetyl-CoA and a fatty acyl-CoA shortened by two carbon atoms [53], also promotes the differentiation of sheep precursor adipocytes into adipocytes [54]. These previous findings, along with our results, confirm the crucial role of these genes and the β-oxidation pathway in the control of fat deposition in this crossbreed.

In addition to the key genes enriched in the two pathways, we also identified several other candidate genes that are involved in fat deposition, such as *LPL*, *ACSM5*, *GK*, and so on. LPL catalyzes the hydrolysis of triglycerides in lipoproteins to release fatty acids for cellular uptake. Comparative transcriptome analysis from castrated cattle showed its abnormal expression during increases in IMF [55]. *ACSM5*, a gene involved in the activation of medium-chain fatty acids, was previously identified by eGWAS as a potential candidate affecting porcine meat quality [56]. *GK*, which was more abundant in chicken breast muscle with higher IMF content [46], was also detected in our study. Although these genes are known to be involved in lipid regulation processes in cattle and other species, and our findings suggest that they may play an important role in IMF control in Wagyu–Angus crossbreeds, a more comprehensive understanding of the specific mechanisms regulating bovine IMF deposition is required.

In this study, we hypothesized a trans-tissue mechanism of IMF deposition in bovine muscle by integrating transcriptomic data obtained from the liver and LD muscle. Briefly, reduced expression of *ACOX2*, *ACOX3*, and *SLC27A1* in the liver inhibited fatty acid uptake and oxidation, resulting in more free fatty acids (FFA) entering the circulation mediated by very low-density lipoproteins (VLDL), which provided sufficient substrate for IMF deposition. Meanwhile, in the LD muscle, the upregulation of *SLC27A1*, *LPL*, and *GK* creates an efficient network for lipid uptake and synthesis: SLC27A1 and LPL promote the transport and supply of fatty acids, while GK accelerates glycerol metabolism and provides the backbone for triglyceride (TAG) synthesis; furthermore, the upregulation of β-oxidation genes (e.g., *CPT1B*) in the muscle tissues may provide more substrates for lipid de novo lipogenesis. Ultimately, the rate of lipid uptake and esterification is greater than the rate of degradation, leading to an accumulation of IMF. This metabolic coupling suggests that IMF deposition in crossbred cattle is driven by preferential shunting of hepatic lipid reserves to muscle, rather than de novo lipogenesis dominance, a finding that contrasts with previous study suggesting that de novo lipogenesis is the primary contributor to IMF [57].

While this study sheds light on the genes and pathways regulating IMF deposition of the crossbred, certain limitations must be acknowledged. Primarily, our study focused on a specific crossbreed, and the genetic architecture of IMF may differ in other cattle breeds. Future studies should include purebred animals and other crossbred combinations to assess the broader relevance of our findings and to identify breed-specific regulators of IMF deposition. Furthermore, the precise nature of hepatic-muscle communication signals, such as exosomes or hormones, remains to be fully elucidated. Moreover, the mechanistic resolution of our study is currently confined to the mRNA level. To address these limitations, future research should integrate spatial transcriptomics and metabolic flux analysis to dynamically characterize the spatial and temporal aspects of lipid partitioning. Finally, functional validation of the identified candidate genes through techniques such as gene knockout or overexpression experiments is necessary to confirm their roles in IMF regulation.

## 5. Conclusions

In conclusion, this study utilized RNA-Seq to investigate the genetic mechanisms underlying IMF deposition in Wagyu–Angus crossbred F1 bulls, a prevalent approach to enhance marbling traits in China. Comparative transcriptomic analysis identified the involvement of key lipid metabolic pathways, including fatty acid beta-oxidation and PPAR signaling, with 11 candidate genes (*ACAA2*, *ACADL*, *ACOX2*, *CPT1B*, *CPT2*, *LPL*, *SLC27A1*, *ACAT1*, *GK*, *ACOX3*, and *ACSM5*) were identified as potential regulators of IMF deposition. We also initially constructed a “liver–muscle” regulatory network to promote IMF deposition. These findings provide novel molecular markers for enhancing IMF content and accelerating genetic selection programs aimed at improving beef quality traits in Wagyu–Angus crossbred cattle. Future research should focus on the following: (1) examining breed-specific genetic factors in IMF deposition by including diverse cattle breeds; (2) elucidating hepatic-muscle communication mechanisms; and (3) functionally validating candidate genes across diverse genetic backgrounds. These efforts will clarify the universality of our findings and refine marker-assisted selection strategies.

## Figures and Tables

**Table 1:** Carcass and meat quality traits of high and low groups.

Traits	High IMF Group	Low IMF Group	*p*
Carcass weight/kg	421.50 ± 44.16	425.00 ± 55.24	0.924
IMF/%	29.02 ± 6.28	11.70 ± 1.10	0.010
Fat color	2.00 ± 0.00	2.50 ± 0.58	0.134
Meat color	2.00 ± 0.00	3.00 ± 0.00	-
Marbling level	4.25 ± 0.50	1.75 ± 0.50	0.000

## Data Availability

The original contributions presented in the study are publicly available. All sequencing data are available through the NCBI Sequence Read Archive under the accession number PRJNA1225315.

## Supplementary Materials

The following supporting information can be downloaded at: https://www.mdpi.com/article/10.3390/ani15091306/s1, Table S1: The primer information of qRT-PCR; Table S2: Statistics of sequencing data; Table S3: DEGs of LL vs. HL group; Table S4: DEGs of the LM vs. HM group; Table S5: Co-DEGs between the liver and muscle; Table S6: Significantly enriched GO terms of DEGs from LL vs. HL group; Table S7: Significantly enriched GO terms of DEGs from LM vs. HM group; Table S8: Significantly enriched KEGG pathways of DEGs from LL vs. HL group; Table S9: Significantly enriched KEGG pathways of DEGs from the LM vs. HM group; Table S10: Information of hub genes associated with IMF deposition; Table S11. Composition and nutritional levels of the TMR for experimental cattle.

## Abbreviations

The following abbreviations are used in this manuscript:

ABHD1	Abhydrolase Domain Containing 1
ACAA2	Acetyl-CoA Acyltransferase 2
ACADL	Acyl-CoA Dehydrogenase Long Chain
ACAT1	Acetyl-CoA Acetyltransferase 1
ACOT2	Acyl-CoA Thioesterase 2
ACOX2	Acyl-CoA Oxidase 2
ACOX3	Acyl-CoA Oxidase 3
ACSL4	Acyl-CoA Synthetase Long Chain Family Member 4
ACSM5	Acyl-CoA Synthetase Medium Chain Family Member 5
ACTB	Actin Beta
AdipoQ	Adiponectin
C/EBP	CCAAT/Enhancer Binding Protein
CD74	CD74 Molecule
CPT1B	Carnitine Palmitoyltransferase 1B
CPT2	Carnitine Palmitoyltransferase 2
CXCL10	C-X-C Motif Chemokine Ligand 10
DGAT2	Diacylglycerol O-Acyltransferase 2
EBPL	Emopamil Binding Protein Like
EGR1	Early Growth Response 1
EPHX2	Epoxide Hydrolase 2
FABPs	Fatty Acid Binding Proteins
FASN	Fatty Acid Synthase
FATP1	Fatty Acid Transport Protein 1
GK	Glycerol Kinase
GPAM	Glycerol-3-Phosphate Acyltransferase
HK2	Hexokinase 2
HRG	Heregulin
IGFBP1	Insulin Like Growth Factor Binding Protein 1
IRF7	Interferon Regulatory Factor 7
ITGB1	Integrin Subunit Beta 1
LIPE	Lipase E, Hormone Sensitive Type
LPL	Lipoprotein Lipase
MC4R	Melanocortin 4 Receptor
MYF5	Myogenic Factor 5
NAIP	NLR Family Apoptosis Inhibitory Protein
NCAM1	Neural Cell Adhesion Molecule 1
PARP14	Poly(ADP-Ribose) Polymerase 14
PLAC8A	Placenta Specific 8A
PLTP	Phospholipid Transfer Protein
PPAR	Peroxisome Proliferator Activated Receptor
PTGS1	Prostaglandin-Endoperoxide Synthase 1
PTGS2	Prostaglandin-Endoperoxide Synthase 2
RAR	Retinoic Acid Receptor
RET	Ret Proto-Oncogene
REXO2	RNA exonuclease 2
SCD	Stearoyl-CoA Desaturase
SLC27A1	Solute Carrier Family 27 Member 1
SREBP	Sterol Regulatory Element Binding Protein
